# Supplementary figures and images for: Conformational dynamics of auto-inhibition in the ER calcium sensor STIM1
Source: eLife. 2021 Nov 3;10:e66194. doi: 10.7554/eLife.66194 (PMC8651296; doi:10.7554/eLife.66194)

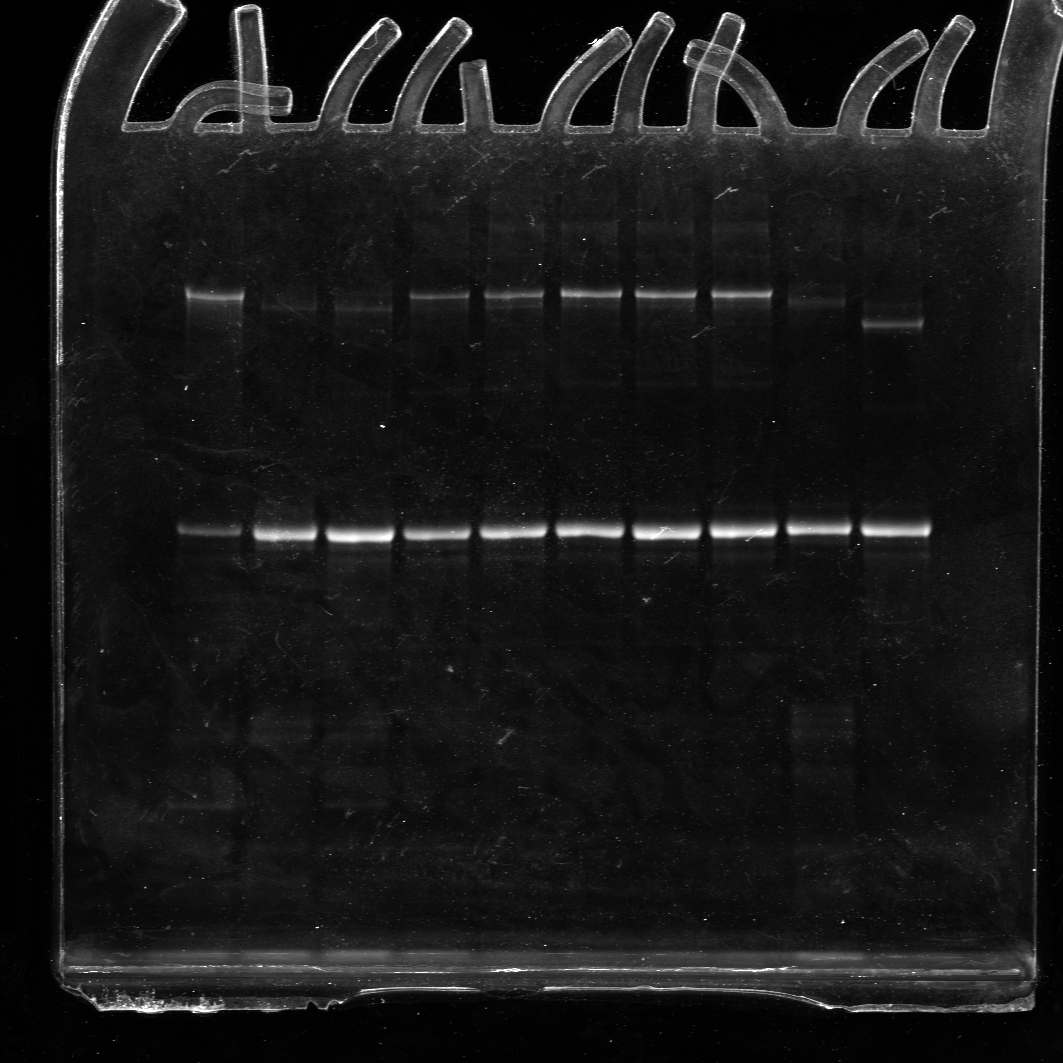

Supplement: Figure 2—figure supplement 1—source data 1. [file elife-66194-fig2-figsupp1-data1.zip › Figure 2 - figure supplement 1 - source data 1.TIF]

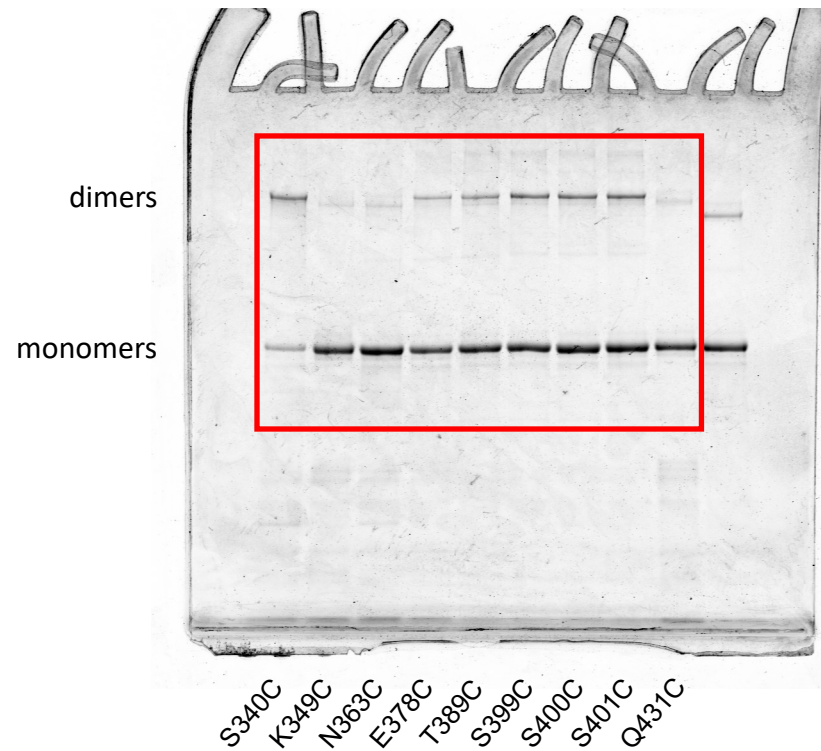

Supplement: Figure 2—figure supplement 1—source data 2. [file elife-66194-fig2-figsupp1-data2.zip › Figure 2 - figure supplement 1 - source data 2.pdf]

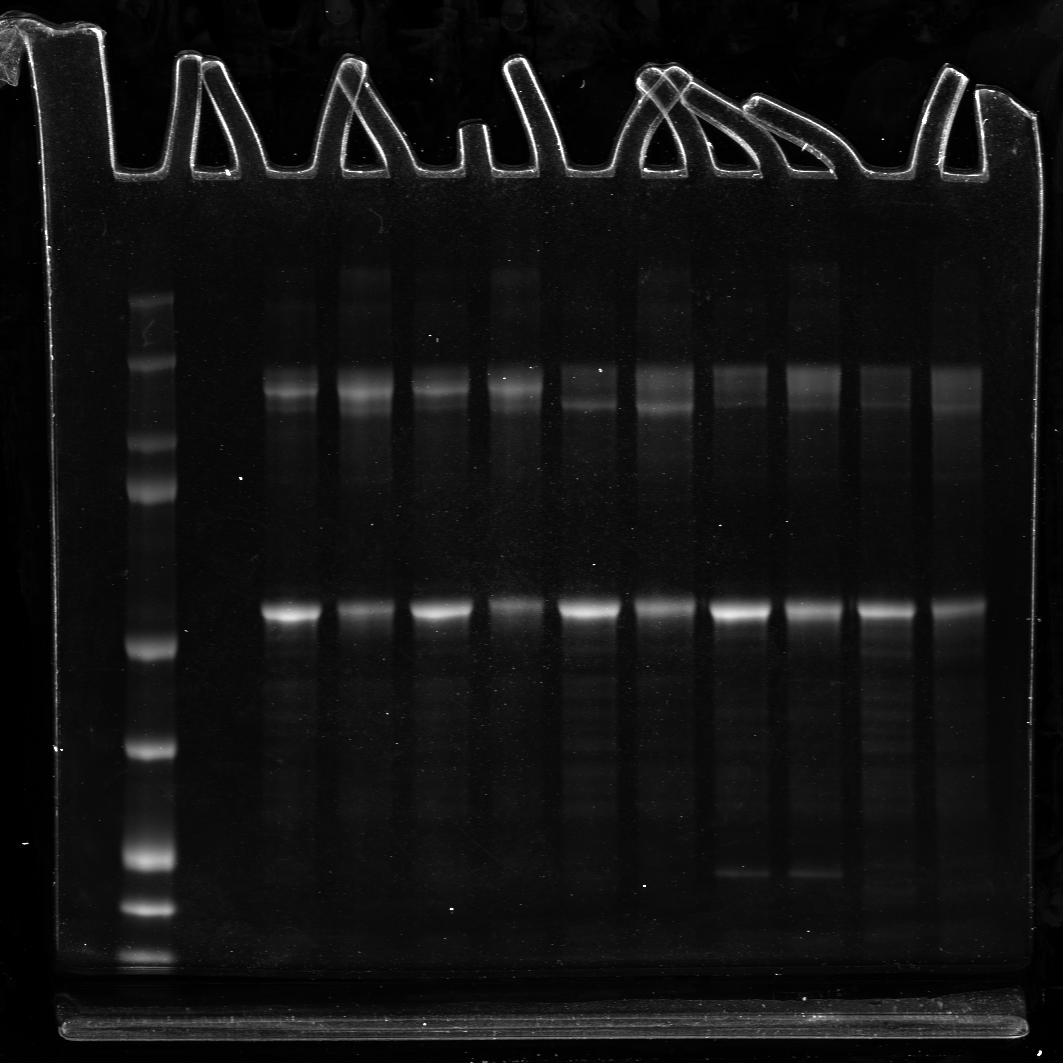

Supplement: Figure 5—figure supplement 2—source data 1. [file elife-66194-fig5-figsupp2-data1.zip › Figure 5 - figure supplement 2 - source data 1.TIF]

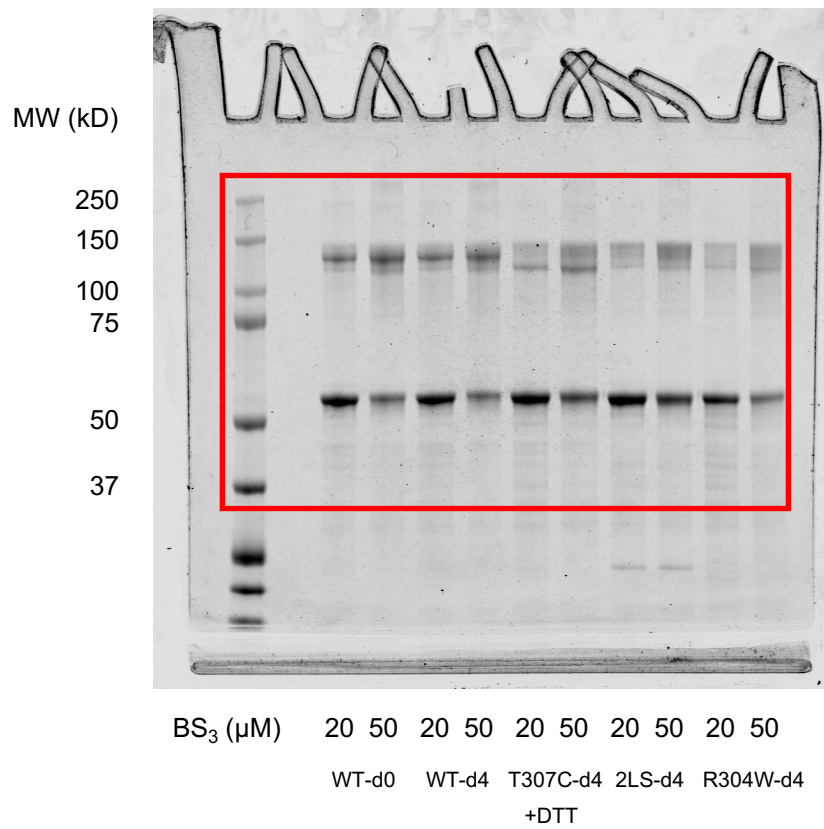

Supplement: Figure 5—figure supplement 2—source data 2. [file elife-66194-fig5-figsupp2-data2.zip › Figure 5 - figure supplement 2 - source data 2.pdf]

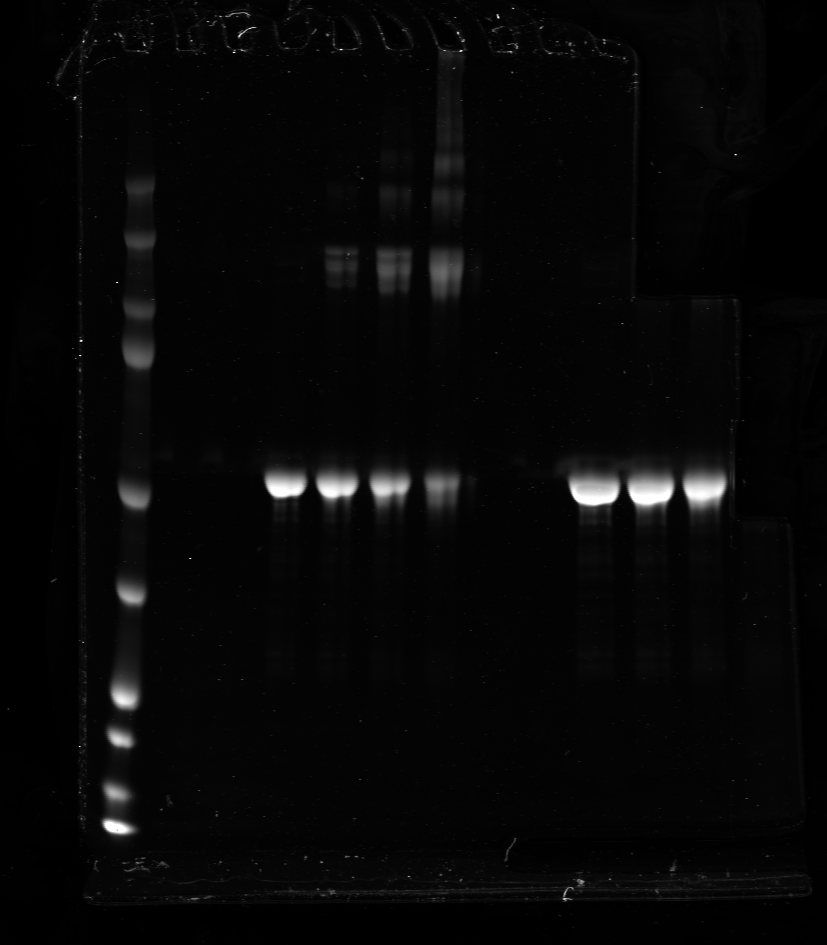

Supplement: Figure 5—figure supplement 3—source data 1. [file elife-66194-fig5-figsupp3-data1.zip › Figure 5 - figure supplement 3 - source data 1.TIF]

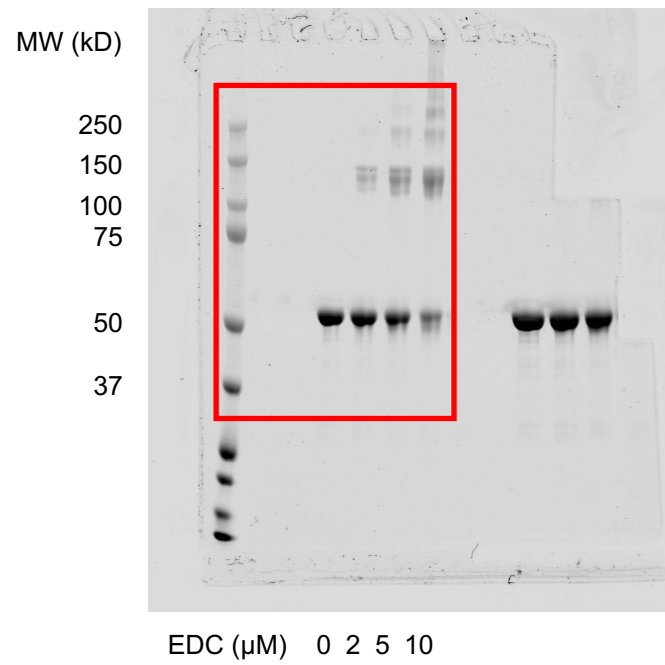

Supplement: Figure 5—figure supplement 3—source data 2. [file elife-66194-fig5-figsupp3-data2.zip › Figure 5 - figure supplement 3 - source data 2.pdf]

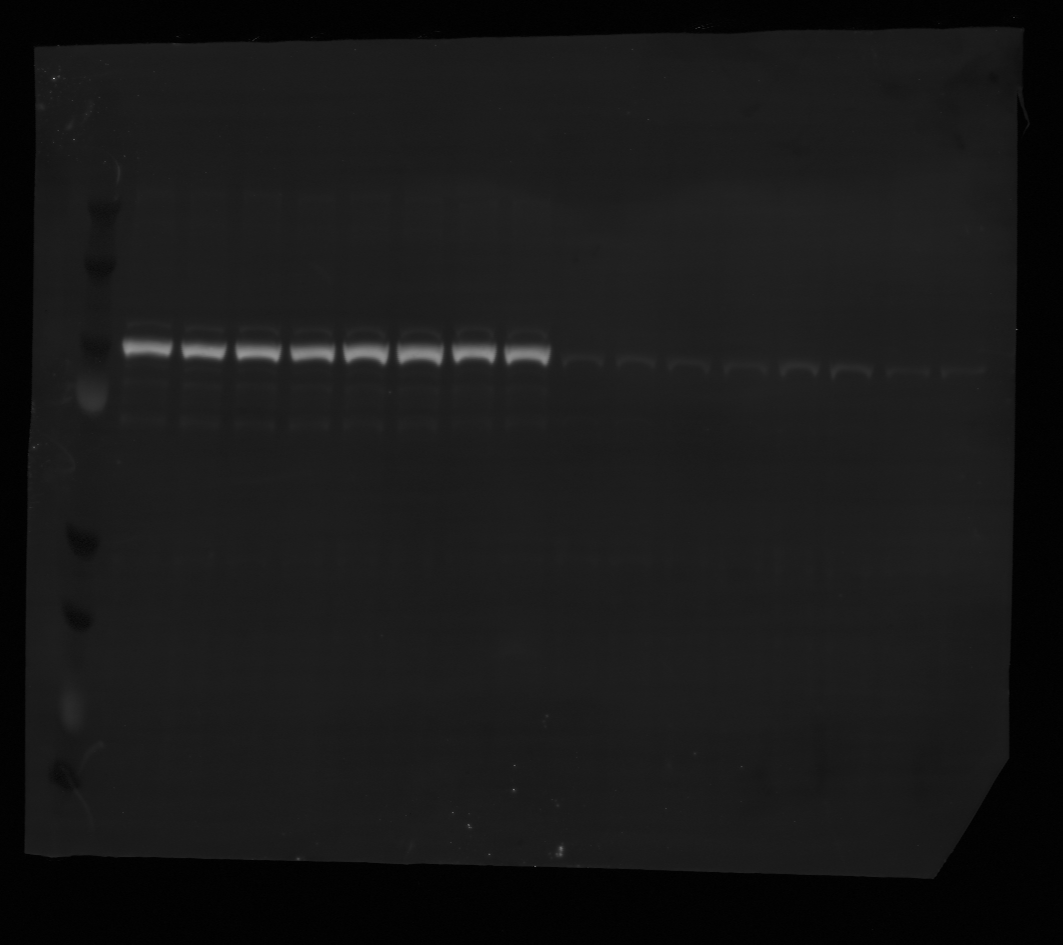

Supplement: Figure 7—source data 1. [file elife-66194-fig7-data1.zip › Figure 7 - source data 1 /Figure 7 - raw unedited western blots/Figure 7 - raw unedited western blots_WT.TIF]

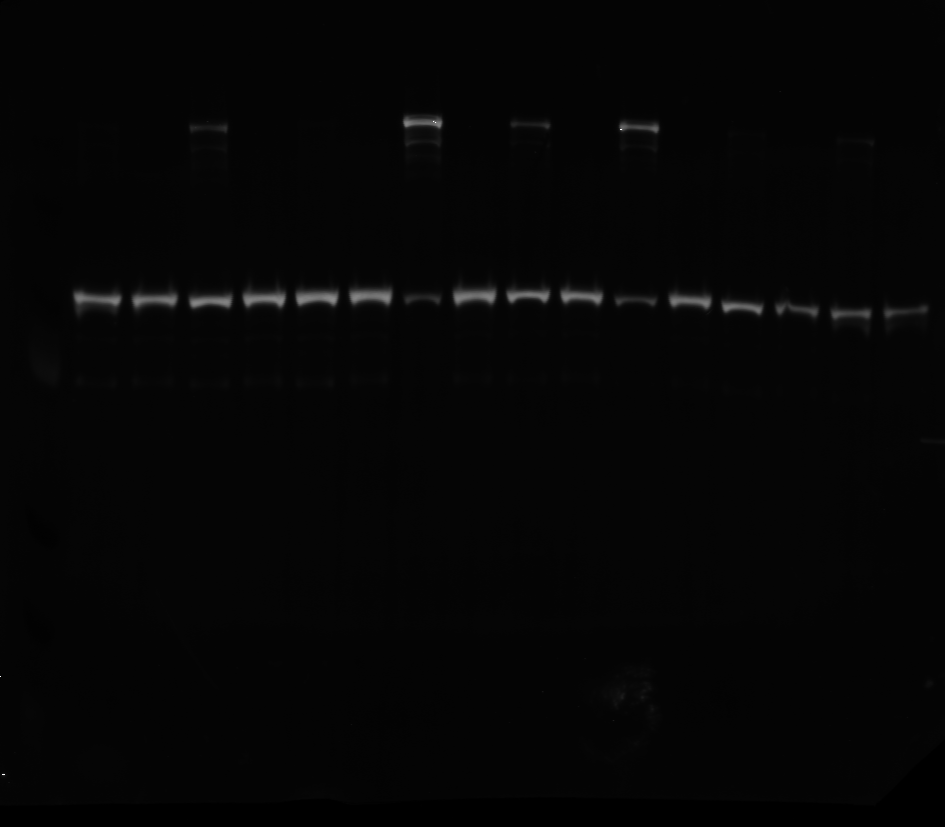

Supplement: Figure 7—source data 1. [file elife-66194-fig7-data1.zip › Figure 7 - source data 1 /Figure 7 - raw unedited western blots/Figure 7 - raw unedited western blots_A268C and T307C.TIF]

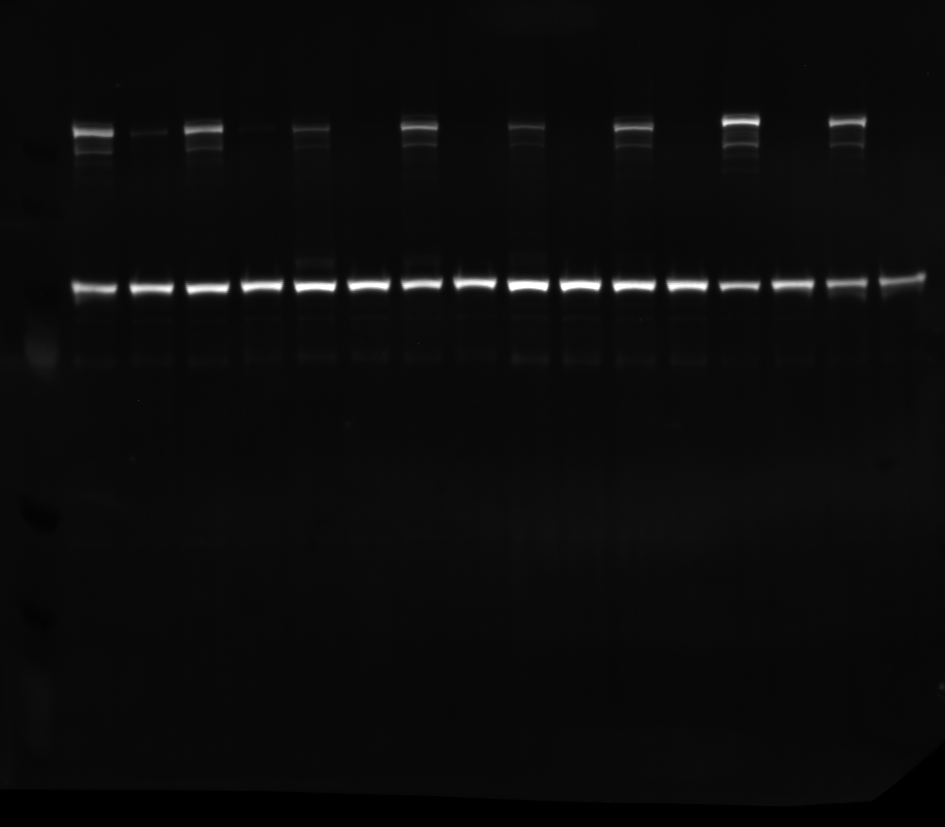

Supplement: Figure 7—source data 1. [file elife-66194-fig7-data1.zip › Figure 7 - source data 1 /Figure 7 - raw unedited western blots/Figure 7 - raw unedited western blots_S339C.TIF]

Figure 7A- S339C

| Lane    | 14    | 15 | 16         | 17 |
|---------|-------|----|------------|----|
| Sample  | S339C |    |            |    |
| Soln    | 2 Ca  |    | 0 Ca + CPA |    |
| Diamide | +     | +  | +          | +  |
| DTT     | -     | +  | -          | +  |

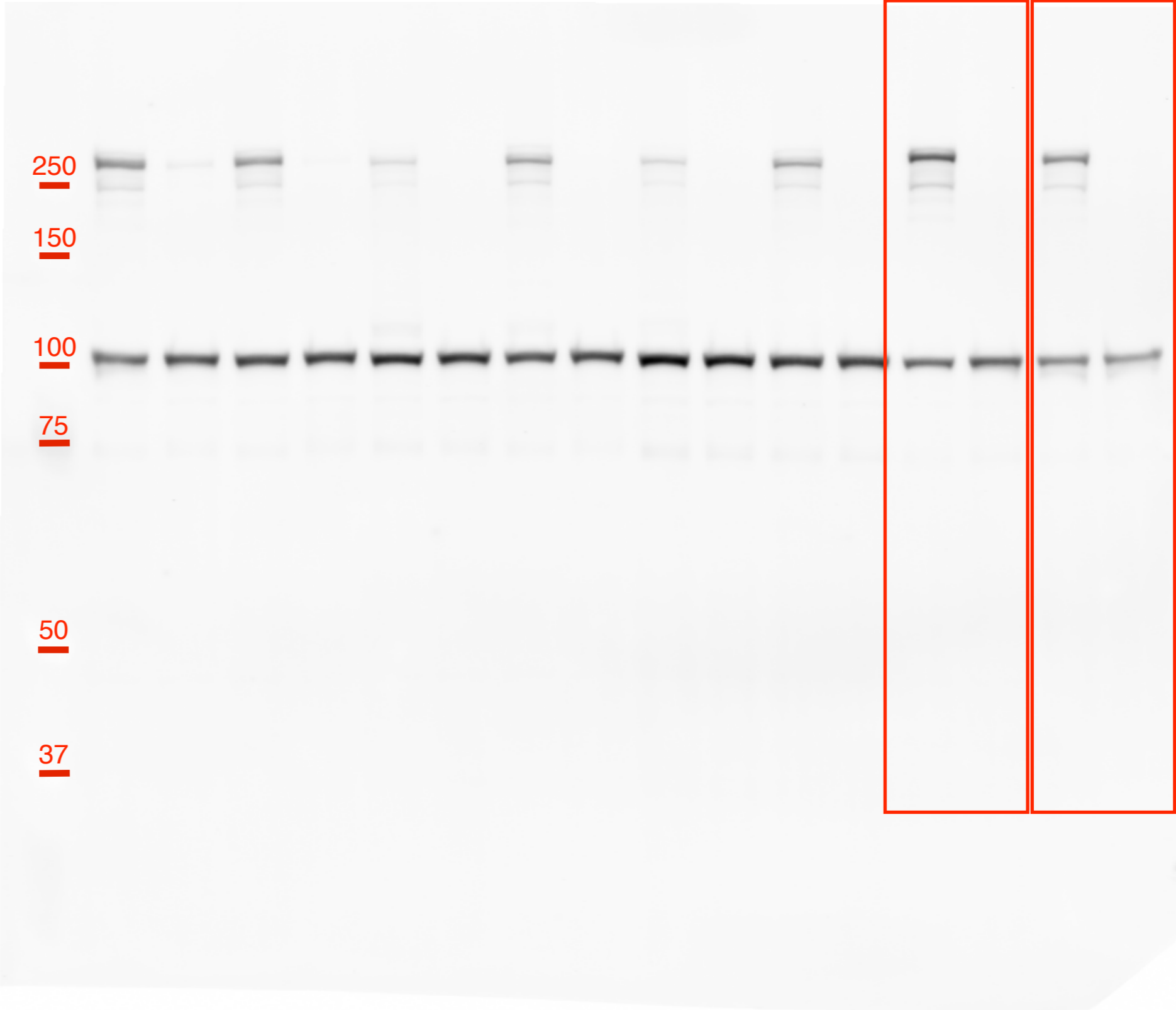

Supplement: Figure 7—source data 1. [file elife-66194-fig7-data1.zip › Figure 7 - source data 1 /Figure 7 - uncropped labeled western blots/Figure 7 - uncropped labeled western blots_S339C.pdf]

Figure 7A- A268C  
and T307C

|         |   |       |   |   |   |            |   |   |   |       |    |            |    |
|---------|---|-------|---|---|---|------------|---|---|---|-------|----|------------|----|
| Lane    | 1 | 2     | 3 | 4 | 5 | 6          | 7 | 8 | 9 | 10    | 11 | 12         | 13 |
| Sample  | M | A268C |   |   |   |            |   |   |   | T307C |    |            |    |
| Soln    |   | 2 Ca  |   |   |   | 0 Ca + CPA |   |   |   | 2 Ca  |    | 0 Ca + CPA |    |
| Diamide |   | -     | - | + | + | -          | - | + | + | +     | +  | +          | +  |
| DTT     |   | -     | + | - | + | -          | + | - | + | -     | +  | -          | +  |

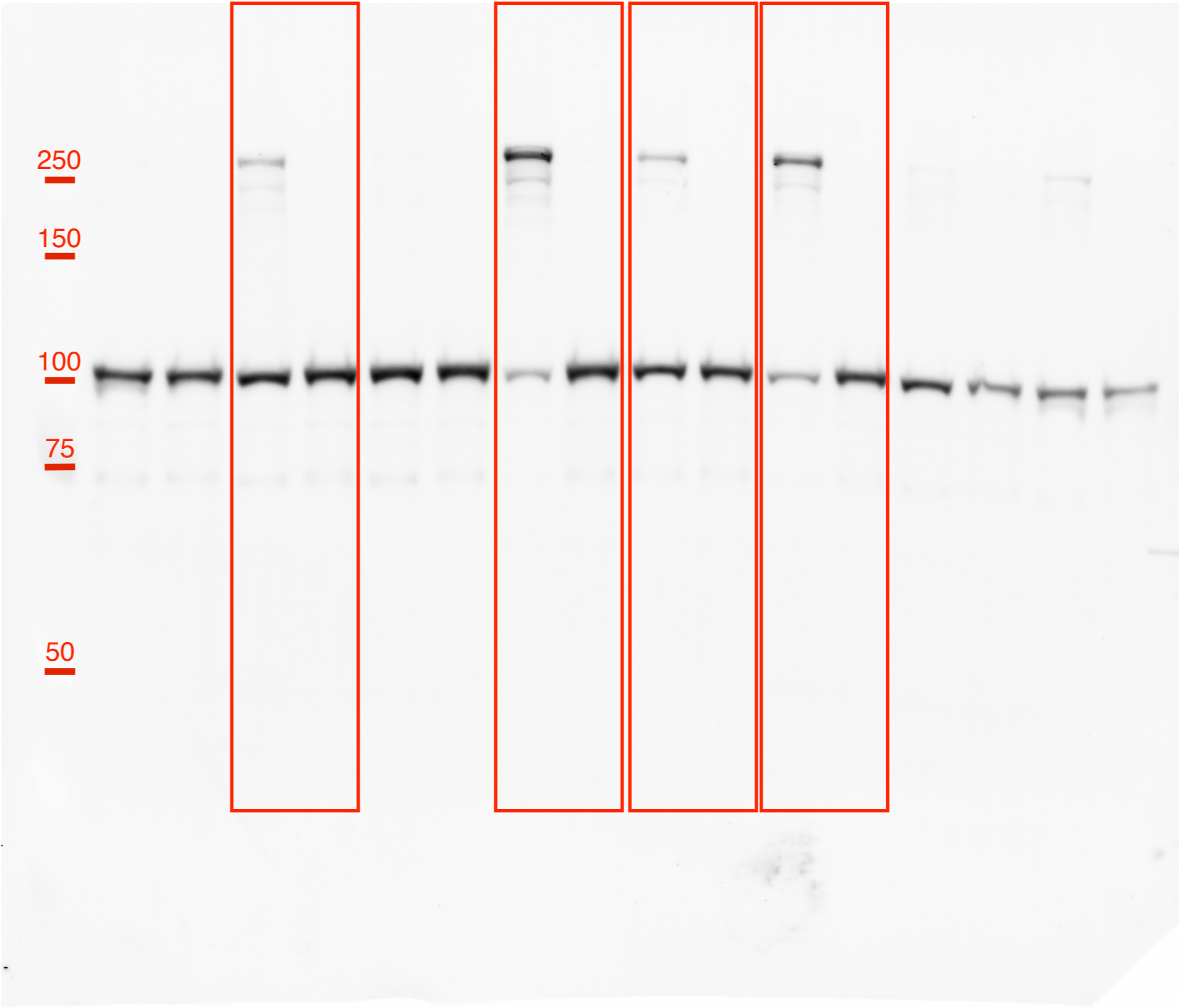

Supplement: Figure 7—source data 1. [file elife-66194-fig7-data1.zip › Figure 7 - source data 1 /Figure 7 - uncropped labeled western blots/Figure 7 - uncropped labeled western blots_A268C and T307C.pdf]

Figure 7A- WT

|         |   |      |   |   |   |            |   |   |   |
|---------|---|------|---|---|---|------------|---|---|---|
| Lane    | 1 | 2    | 3 | 4 | 5 | 6          | 7 | 8 | 9 |
| Sample  | M | WT   |   |   |   | WT         |   |   |   |
| Soln    |   | 2 Ca |   |   |   | 0 Ca + CPA |   |   |   |
| Diamide |   | -    | - | + | + | -          | - | + | + |
| DTT     |   | -    | + | - | + | -          | + | - | + |

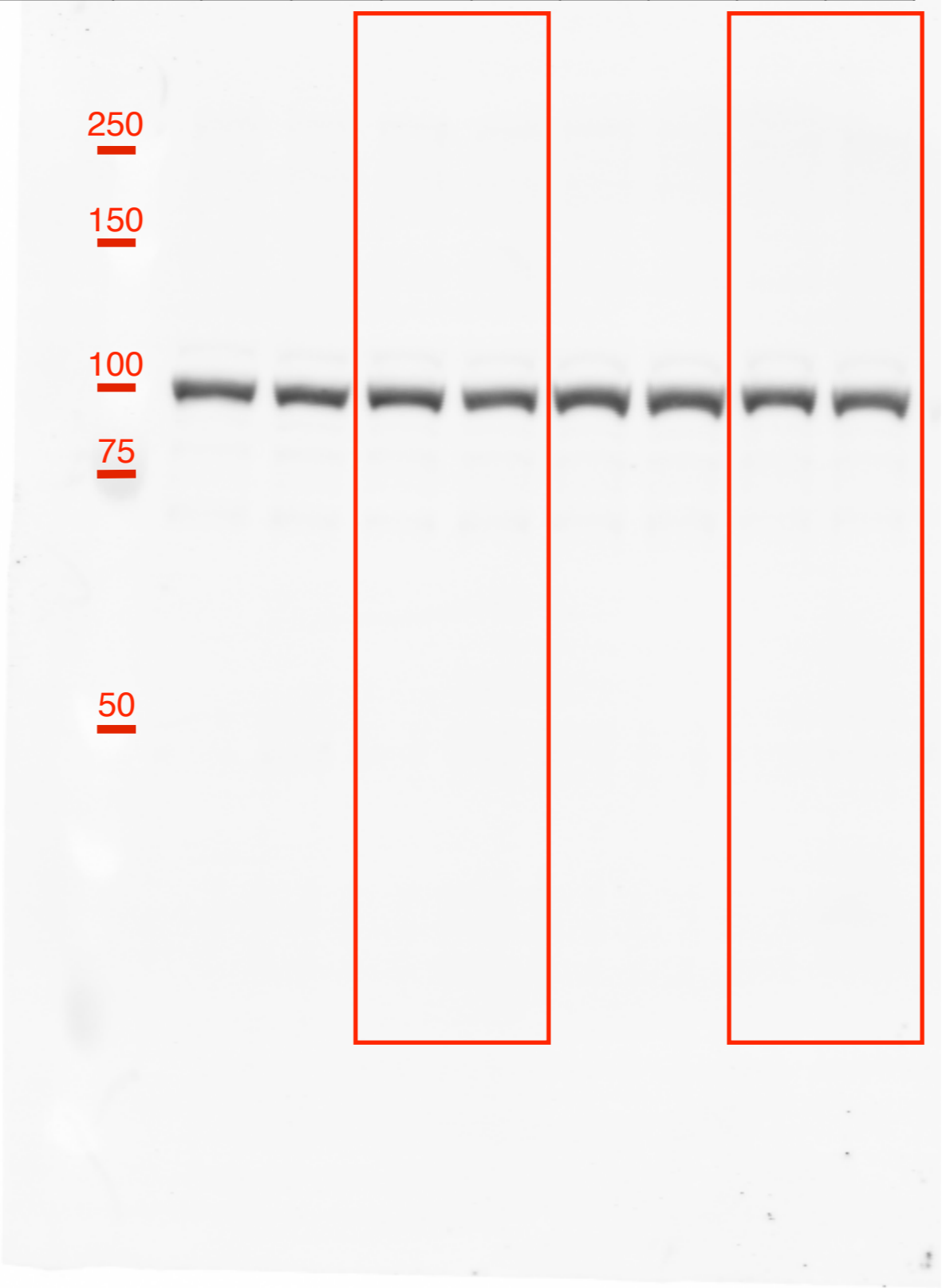

Supplement: Figure 7—source data 1. [file elife-66194-fig7-data1.zip › Figure 7 - source data 1 /Figure 7 - uncropped labeled western blots/Figure 7 - uncropped labeled western blots_WT.pdf]

Figure 7 -  
Supplement 1B  
- N309C

|         |   |       |   |   |   |            |   |   |   |
|---------|---|-------|---|---|---|------------|---|---|---|
| Lane    | 1 | 2     | 3 | 4 | 5 | 6          | 7 | 8 | 9 |
| Sample  | M | N309C |   |   |   |            |   |   |   |
| Soln    |   | 2 Ca  |   |   |   | 0 Ca + CPA |   |   |   |
| Diamide |   | -     | - | + | + | -          | - | + | + |
| DTT     |   | -     | + | - | + | -          | + | - | + |

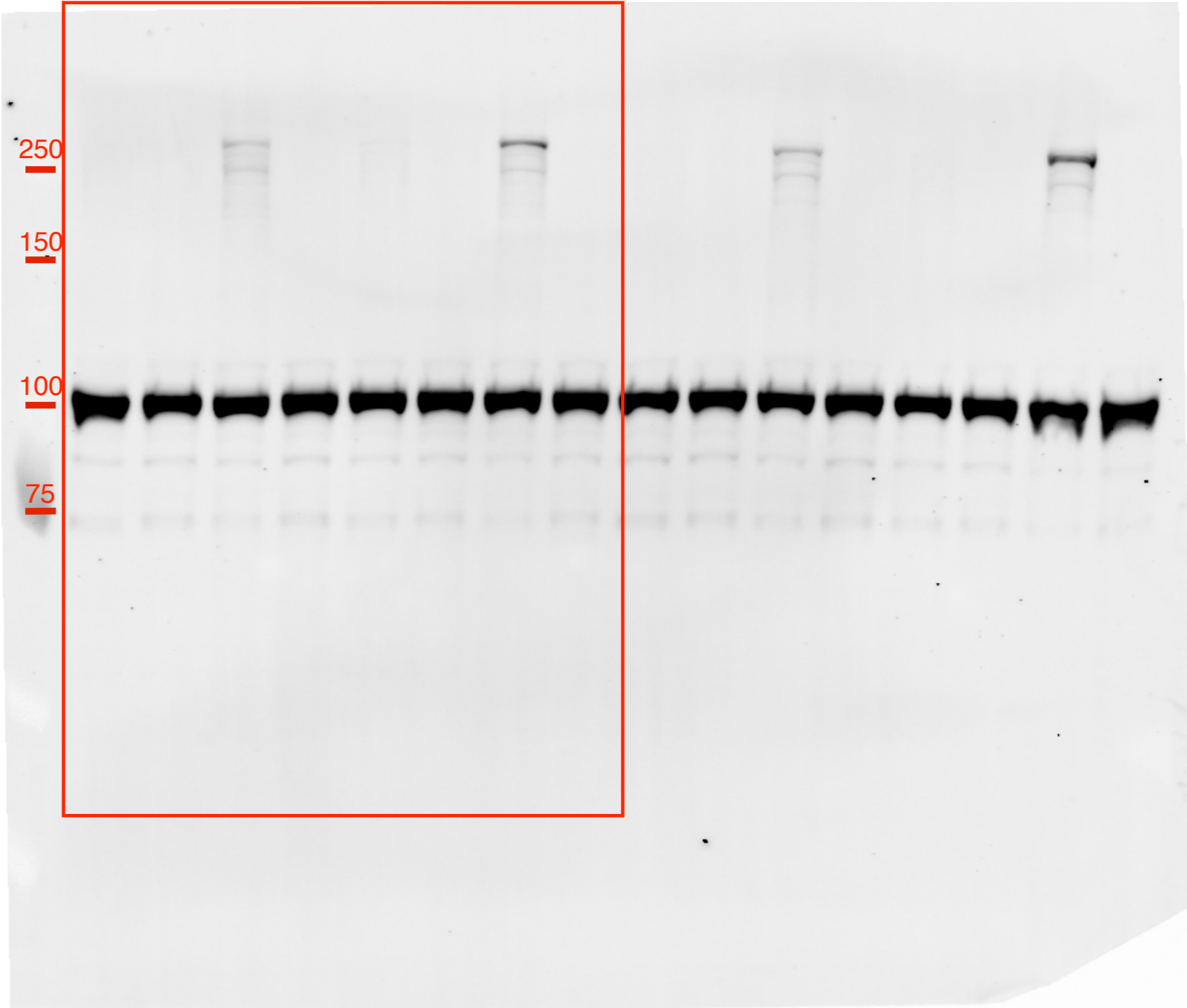

Supplement: Figure 7—figure supplement 1—source data 1. [file elife-66194-fig7-figsupp1-data1.zip › Figure 7 - figure supplement 1 - source data 1/Figure 7 - figure supplement 1 - uncropped labeled gel and western blots/Figure 7 ΓÇô figure supplement 1 ΓÇô panel B_N309C.pdf]

Figure 7 -  
Supplement 1B  
- A268C

|         |   |       |   |   |   |            |   |   |   |
|---------|---|-------|---|---|---|------------|---|---|---|
| Lane    | 1 | 2     | 3 | 4 | 5 | 6          | 7 | 8 | 9 |
| Sample  | M | A268C |   |   |   |            |   |   |   |
| Soln    |   | 2 Ca  |   |   |   | 0 Ca + CPA |   |   |   |
| Diamide |   | -     | - | + | + | -          | - | + | + |
| DTT     |   | -     | + | - | + | -          | + | - | + |

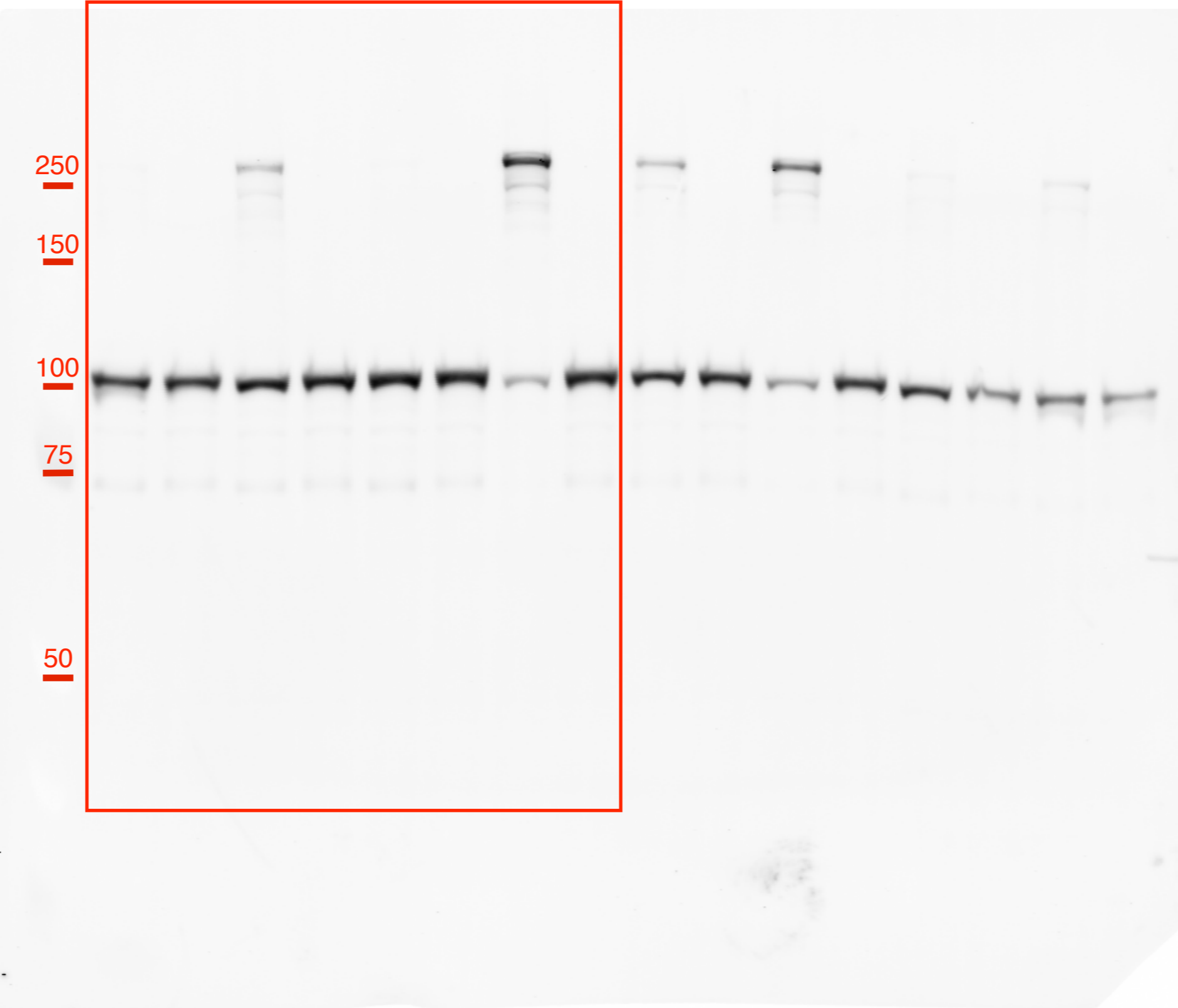

Supplement: Figure 7—figure supplement 1—source data 1. [file elife-66194-fig7-figsupp1-data1.zip › Figure 7 - figure supplement 1 - source data 1/Figure 7 - figure supplement 1 - uncropped labeled gel and western blots/Figure 7 ΓÇô figure supplement 1 ΓÇô panel B_A268C.pdf]

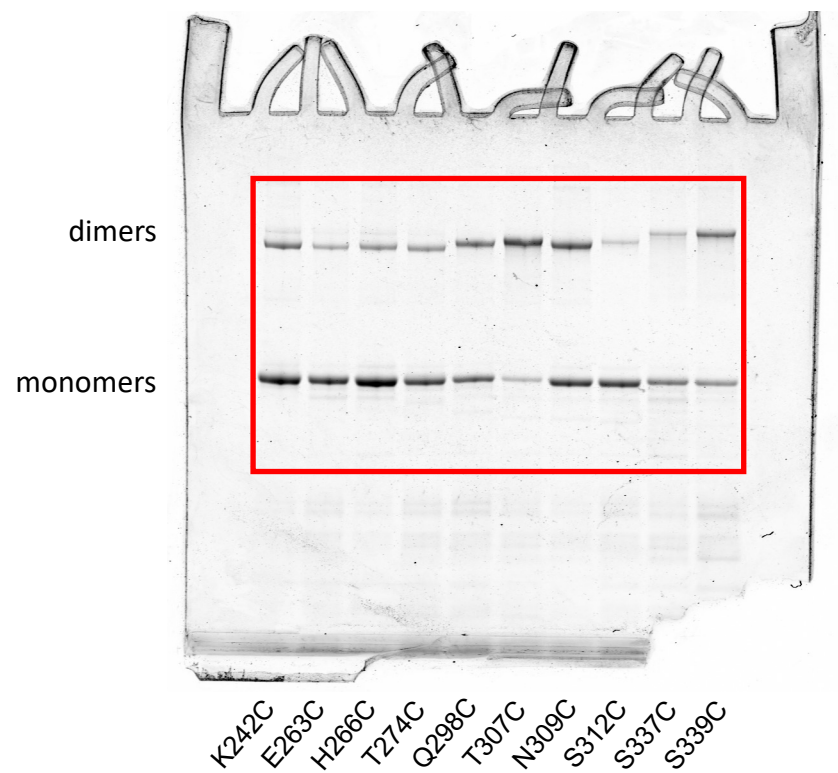

Supplement: Figure 7—figure supplement 1—source data 1. [file elife-66194-fig7-figsupp1-data1.zip › Figure 7 - figure supplement 1 - source data 1/Figure 7 - figure supplement 1 - uncropped labeled gel and western blots/Figure 7 - figure supplement 1 - panel A.pdf]

Figure 7 -  
Supplement 1B  
- T307C

|         |   |   |   |   |   |   |   |   |   |       |    |    |    |            |    |    |    |
|---------|---|---|---|---|---|---|---|---|---|-------|----|----|----|------------|----|----|----|
| Lane    | 1 | 2 | 3 | 4 | 5 | 6 | 7 | 8 | 9 | 10    | 11 | 12 | 13 | 14         | 15 | 16 | 17 |
| Sample  | M |   |   |   |   |   |   |   |   | T307C |    |    |    |            |    |    |    |
| Soln    |   |   |   |   |   |   |   |   |   | 2 Ca  |    |    |    | 0 Ca + CPA |    |    |    |
| Diamide |   |   |   |   |   |   |   |   |   | -     | -  | +  | +  | -          | -  | +  | +  |
| DTT     |   |   |   |   |   |   |   |   |   | -     | +  | -  | +  | -          | +  | -  | +  |

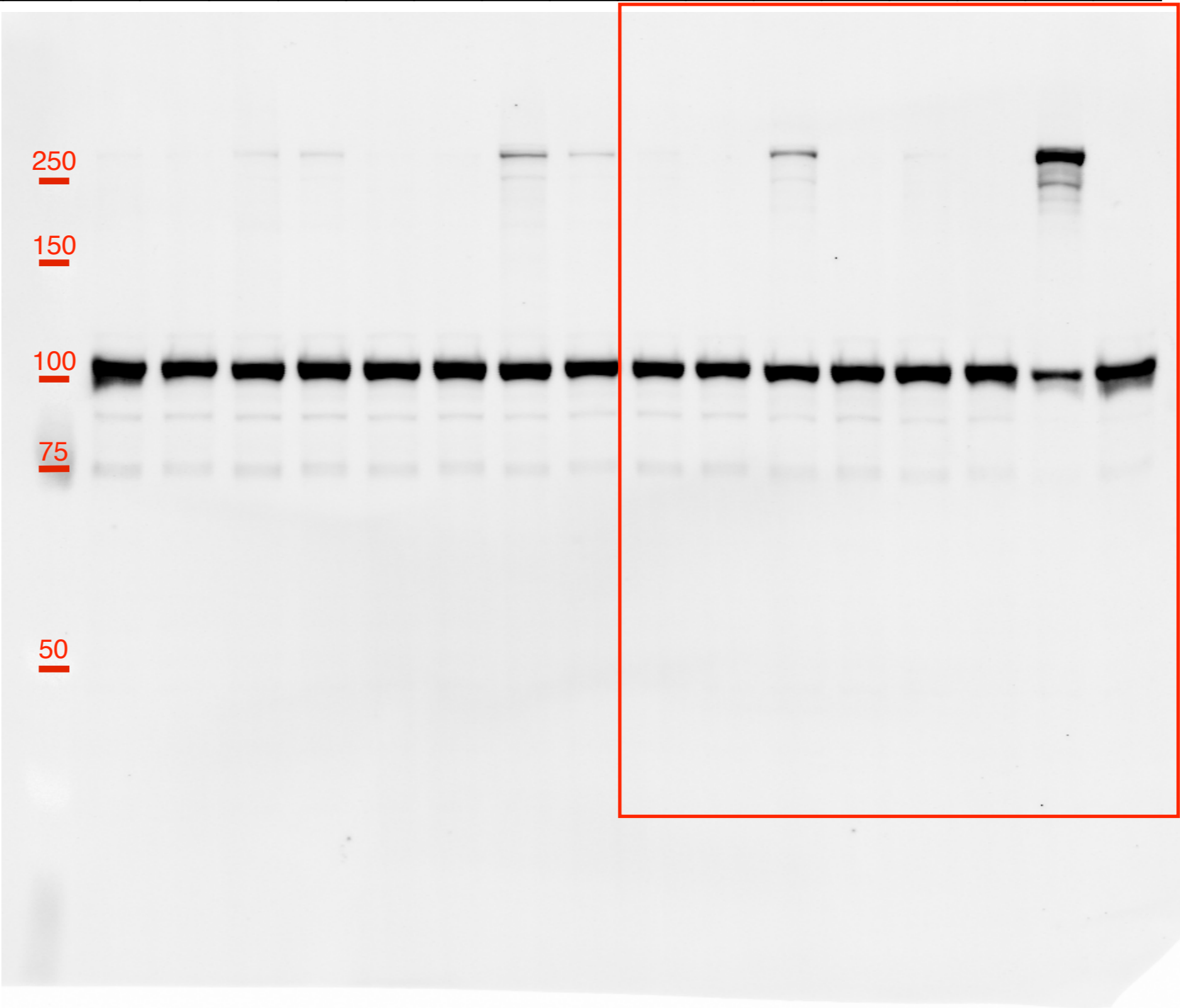

Supplement: Figure 7—figure supplement 1—source data 1. [file elife-66194-fig7-figsupp1-data1.zip › Figure 7 - figure supplement 1 - source data 1/Figure 7 - figure supplement 1 - uncropped labeled gel and western blots/Figure 7 ΓÇô figure supplement 1 ΓÇô panel B_T307C.pdf]

Figure 7 -  
Supplement 1B  
- H266C

|         |   |   |   |   |   |   |   |   |   |       |    |    |    |            |    |    |    |
|---------|---|---|---|---|---|---|---|---|---|-------|----|----|----|------------|----|----|----|
| Lane    | 1 | 2 | 3 | 4 | 5 | 6 | 7 | 8 | 9 | 10    | 11 | 12 | 13 | 14         | 15 | 16 | 17 |
| Sample  | M |   |   |   |   |   |   |   |   | H266C |    |    |    |            |    |    |    |
| Soln    |   |   |   |   |   |   |   |   |   | 2 Ca  |    |    |    | 0 Ca + CPA |    |    |    |
| Diamide |   |   |   |   |   |   |   |   |   | -     | -  | +  | +  | -          | -  | +  | +  |
| DTT     |   |   |   |   |   |   |   |   |   | -     | +  | -  | +  | -          | +  | -  | +  |

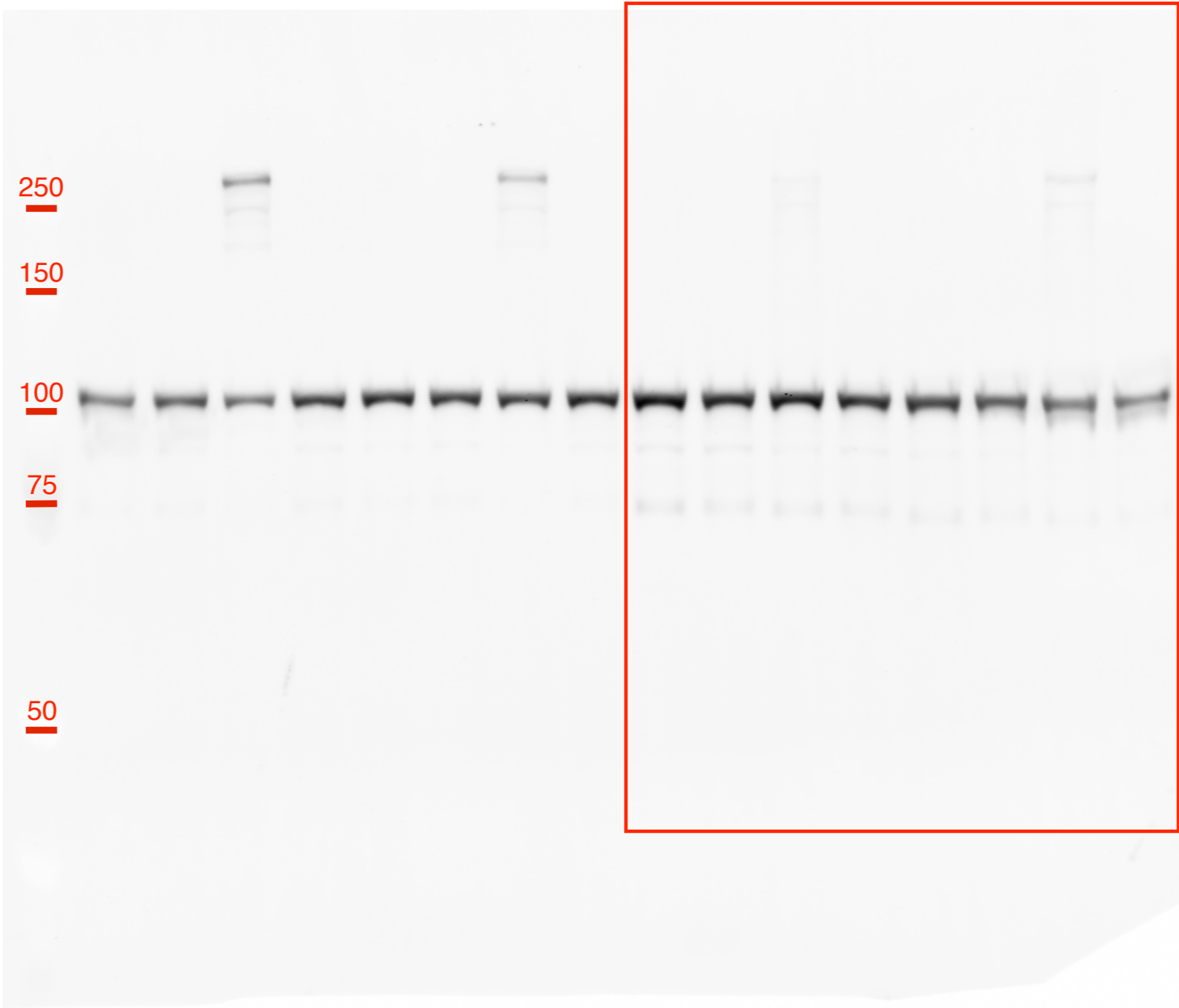

Supplement: Figure 7—figure supplement 1—source data 1. [file elife-66194-fig7-figsupp1-data1.zip › Figure 7 - figure supplement 1 - source data 1/Figure 7 - figure supplement 1 - uncropped labeled gel and western blots/Figure 7 ΓÇô figure supplement 1 ΓÇô panel B_H266C.pdf]

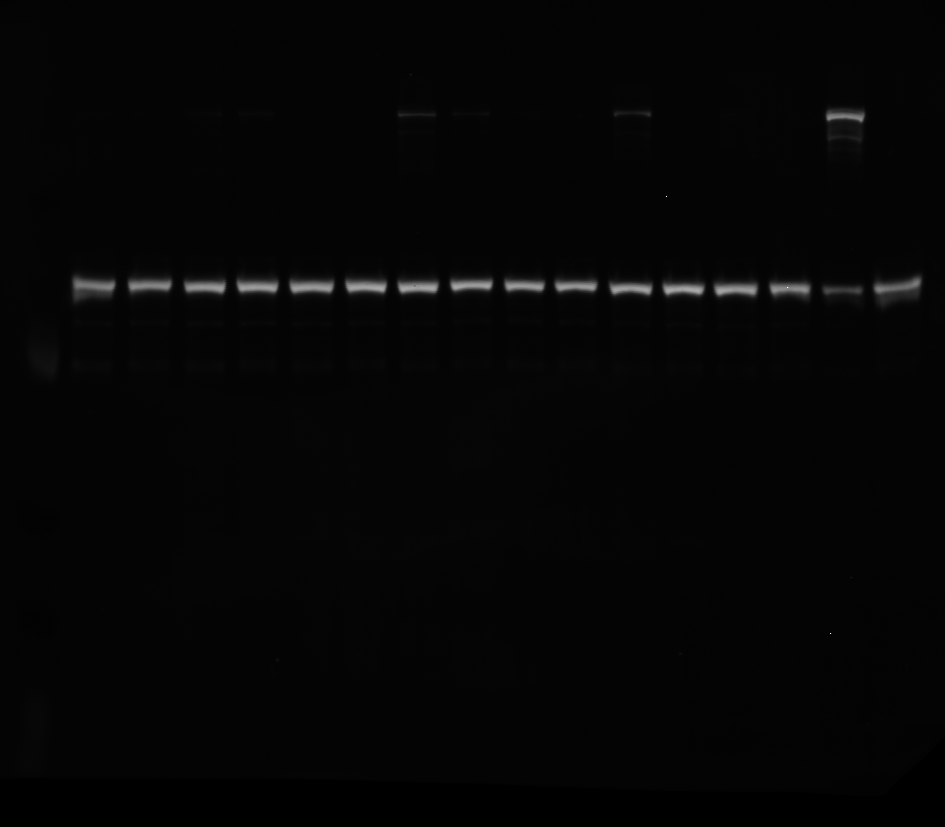

Supplement: Figure 7—figure supplement 1—source data 1. [file elife-66194-fig7-figsupp1-data1.zip › Figure 7 - figure supplement 1 - source data 1/Figure 7 - figure supplement 1 - raw unedited gel and western blots/Figure 7 ΓÇô figure supplement 1 ΓÇô panel B_T307C.TIF]

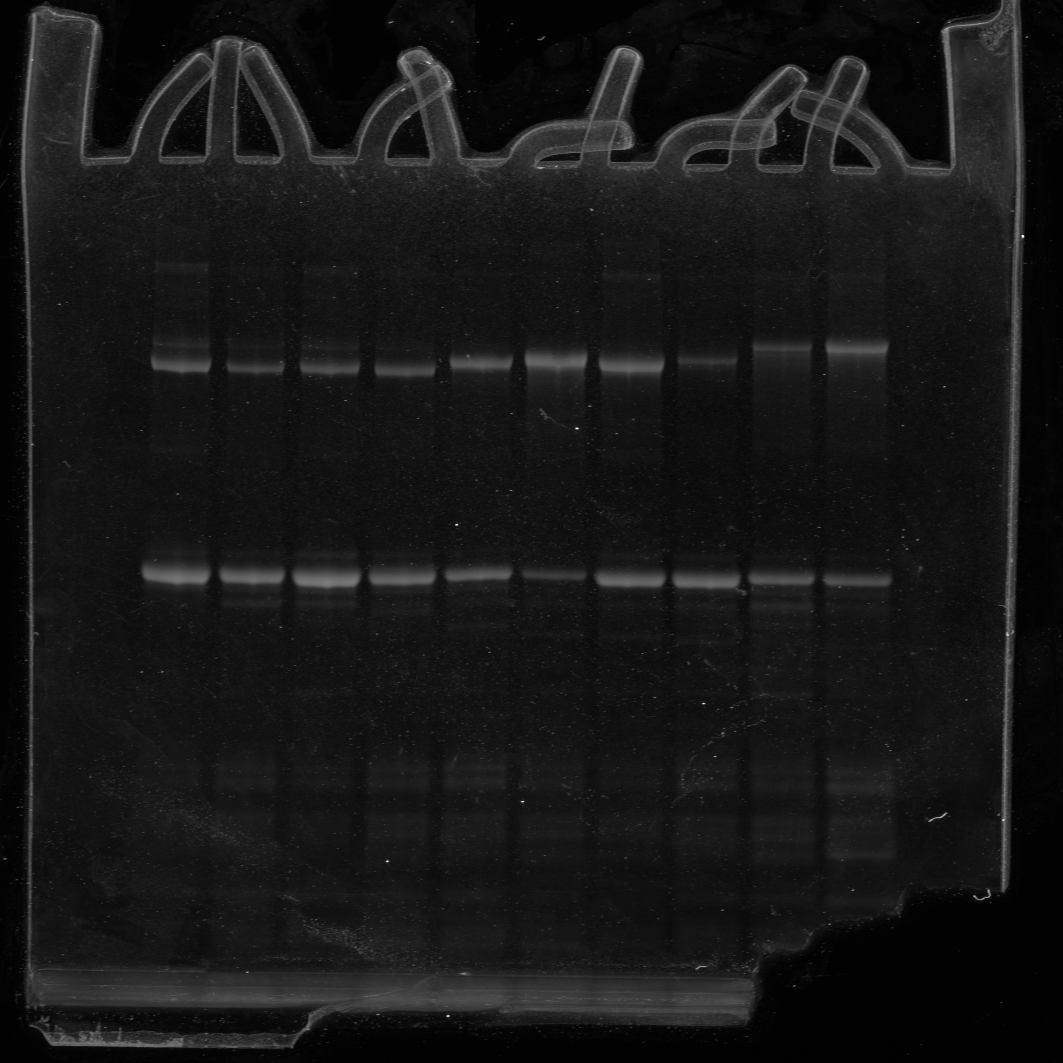

Supplement: Figure 7—figure supplement 1—source data 1. [file elife-66194-fig7-figsupp1-data1.zip › Figure 7 - figure supplement 1 - source data 1/Figure 7 - figure supplement 1 - raw unedited gel and western blots/Figure 7 - figure supplement 1 - panel A.TIF]

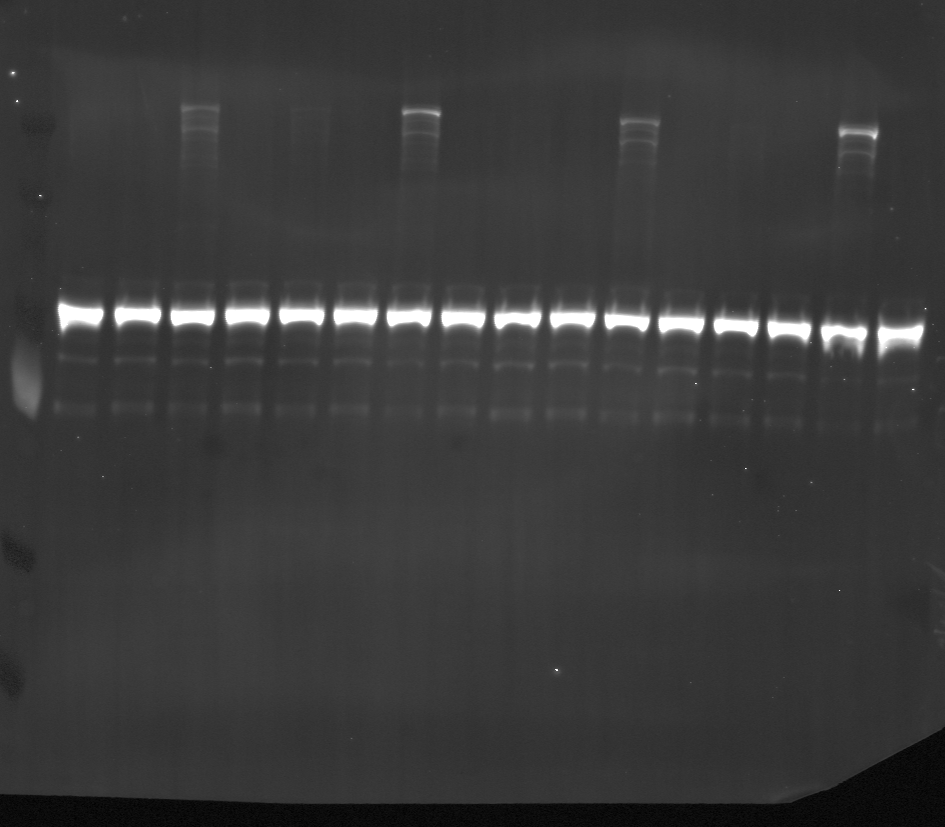

Supplement: Figure 7—figure supplement 1—source data 1. [file elife-66194-fig7-figsupp1-data1.zip › Figure 7 - figure supplement 1 - source data 1/Figure 7 - figure supplement 1 - raw unedited gel and western blots/Figure 7 ΓÇô figure supplement 1 ΓÇô panel B_N309C.TIF]

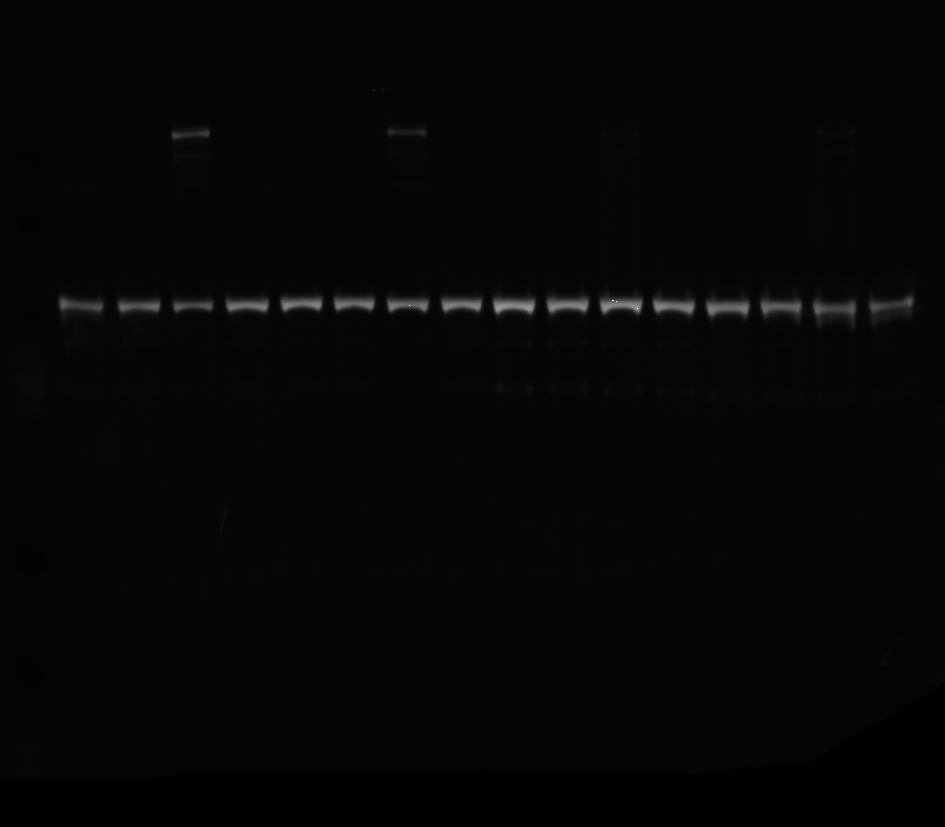

Supplement: Figure 7—figure supplement 1—source data 1. [file elife-66194-fig7-figsupp1-data1.zip › Figure 7 - figure supplement 1 - source data 1/Figure 7 - figure supplement 1 - raw unedited gel and western blots/Figure 7 ΓÇô figure supplement 1 ΓÇô panel B_H266C.TIF]
