# Supplementary material for: Conformational dynamics of auto-inhibition in the ER calcium sensor STIM1
Source: eLife. 2021 Nov 3;10:e66194. doi: 10.7554/eLife.66194 (PMC8651296; doi:10.7554/eLife.66194)
Supplement: Supplementary file 1. — The table lists the complete set of amino acid pairs, their predominant smFRET efficiencies, the corresponding smFRET-derived distances, and the range of distance values used for generating the models shown in Figure 5A and Figure 5—figure supplement 1. The 'FRET peak' and 'Distance' values correspond to the peaks of the smFRET histograms and the associated calculated distances shown in Figure 1—figure supplements 1–3. For sites with both liposome and avitag measurements, the liposome measurement was used to constrain the CC1:CAD model. The ‘Range’ values indicate the allowable distance bounds for each pair of modeled residues (see Materials and methods). [file elife-66194-supp1.docx]

|  | **FRET pair** | **FRET peak** | **Distance (Å)** | **Range (Å)** |
| --- | --- | --- | --- | --- |
| 1 | **239:239'** | 0.26 | 60 | 37 - 64 |
| 2 | **239:274** | 0.55 | 49 | 27 - 51 |
| 3 | **239:400** | 0.54 | 49 | 37 – 51 |
| 4 | **242:242'** | 0.31 | 58 | 35 – 61 |
| 5 | **242:363** | 0.56 | 48 | 36 – 50 |
| 6 | **242:378** | 0.67 | 45 | 33 – 47 |
| 7 | **242:389** | 0.45 | 52 | 40 – 54 |
| 8 | **242:400** | 0.64 | 46 | 34 – 48 |
| 9 | **242:400'** | 0.95 | 31 | 0 – 36 |
| 10 | **242:417** | 0.40 | 54 | 42 – 56 |
| 11 | **242:417'** | 0.90 | 35 | 20 – 38 |
| 12 | **242:431** | 0.30 | 58 | 46 – 61 |
| 13 | **242:431'** | 0.73 | 43 | 30 – 45 |
| 14 | **266:266'** | 0.48 | 51 | 29 – 53 |
| 15 | **266:389** | 0.22 | 62 | 49 – 67 |
| 16 | **274:274'** | 0.50 | 51 | 28 – 53 |
| 17 | **274:307** | 0.62 | 47 | 25 – 49 |
| 18 | **274:337** | 0.88 | 36 | 12 – 39 |
| 19 | **274:400** | 0.24 | 61 | 48 – 65 |
| 20 | **274:400'** | 0.35 | 56 | 44 – 69 |
| 21 | **274:417'** | 0.74 | 42 | 30 – 44 |
| 22 | **274:431** | 0.65 | 46 | 34 – 48 |
| 23 | **274:431'** | 0.90 | 35 | 20 – 38 |
| 24 | **298:298'** | 0.67 | 45 | 23 – 47 |
| 25 | **298:363** | 0.28 | 59 | 46 – 72 |
| 26 | **298:378** | 0.15 | 67 | 53 – 94 |
| 27 | **298:389** | 0.11 | 72 | 56 – 114 |
| 28 | **307:337** | 0.63 | 46 | 24 – 48 |
| 29 | **307:400** | 0.15 | 67 | 53 – 114 |
| 30 | **309:309'** | 0.90 | 35 | 10 – 38 |
| 31 | **312:312'** | 0.87 | 37 | 13 – 40 |
| 32 | **337:337'** | 0.89 | 36 | 11 – 39 |
| 33 | **337:363** | 0.70 | 44 | 32 – 46 |
| 34 | **337:378** | 0.43 | 53 | 41 – 65 |
| 35 | **337:389** | 0.18 | 65 | 51 – 90 |
| 36 | **337:431** | 0.79 | 40 | 28 - 43 |
